# Rapid and Specific Detection of *Listeria monocytogenes* With an Isothermal Amplification and Lateral Flow Strip Combined Method That Eliminates False-Positive Signals From Primer–Dimers

**DOI:** 10.3389/fmicb.2019.02959

**Published:** 2020-02-06

**Authors:** Lei Wang, Panpan Zhao, Xinxin Si, Juan Li, Xiaofang Dai, Kunxiao Zhang, Song Gao, Jingquan Dong

**Affiliations:** ^1^Jiangsu Key Laboratory of Marine Biological Resources and Environment, Jiangsu Key Laboratory of Marine Pharmaceutical Compound Screening, Co-Innovation Center of Jiangsu Marine Bio-industry Technology, Jiangsu Ocean University, Lianyungang, China; ^2^College of Veterinary Medicine, Jilin University, Changchun, China; ^3^Wuhan Institute for Food and Cosmetic Control, Wuhan, China; ^4^School of Pharmacy, Jiangsu Ocean University, Lianyungang, China

**Keywords:** *Listeria monocytogenes*, foodborne pathogen, isothermal amplification, recombinase polymerase amplification, lateral flow strip, false positive, primer–dimer

## Abstract

*Listeria monocytogenes* is an important foodborne pathogenic bacterium that is explicitly threatening public health and food safety. Rapid, simple, and sensitive detection methods for this pathogen are of urgent need for the increasing on-site testing demands. Application of the isothermal recombinase polymerase amplification (RPA) and the lateral flow strip (LFS) in the detection is promising for fast speed, high sensitivity, and little dependency on equipment and trained personnel. However, the simplicity comes with an intrinsic and non-negligible risk, the false-positive signals from primer–dimers. In this study, an improved RPA–LFS system was established for detection of *L. monocytogenes* that eliminated false-positive signals from primer–dimers. Primer candidates were carefully selected from the entire *L. monocytogenes* genome sequence and rigorously screened for specific amplifications in PCR and RPA reactions. For the optimal primer pairs, probes that matched the targeted fragment sequences, although had the smallest chance to form cross-dimers with the primers, were designed and screened. The intelligent use of the probe successfully linked the positive signal to the actual amplification product. This RPA–LFS system was highly specific to *L. monocytogenes* and was able to detect as low as 1 colony-forming unit of the bacterium per reaction (50 μl) without DNA purification, or 100 fg of the genomic DNA/50 μl. The amplification could be conducted under the temperature between 37 and 42°C, and the whole detection finished within 25 min. Test of artificially contaminated milk gave 100% accuracy of detection without purification of the samples. Various food samples spiked with 10 colony-forming unit of *L. monocytogenes* per 25 g or 25 ml were successfully detected after an enrichment time period of 6 h. The RPA–LFS system established in this study is a rapid, simple, and specific detection method for *L. monocytogenes* that has eliminated false-positive results from primer–dimers. In addition, this study has set a good example of eliminating the false-positive risk from primer–dimers in isothermal amplification-based detection methods, which is applicable to the development of detection technologies for other pathogens.

## Introduction

*Listeria monocytogenes* is a facultatively anaerobic non-sporulating gram-positive bacterium responsible for listeriosis in humans and animals (Mook et al., 2011). As an important foodborne pathogenic bacterium, it is explicitly threatening public health and has resulted in serious economic losses worldwide (Carpentier and Cerf, 2011; Pisanu et al., 2018). Its strong capacity of growing under unfavorable conditions, such as low temperature, high salt, and extreme pH, makes *L. monocytogenes* a significant infection source that affects almost all kinds of food (Marcus et al., 2009; Allerberger and Wagner, 2010; Chlebicz and Śliżewska, 2018). The infected animals also become carriers that expand the prevalence of its infection (Fthenakis et al., 1998). Absence of *L. monocytogenes* in ready-to-eat food products has been required by the World Health Organization and food regulatory agencies in many countries (Lianou and Sofos, 2007; Al-Nabulsi et al., 2015).

Biochemical identification and molecular detection methods have been used for detecting *L. monocytogenes*. Biochemical identification is a conventional and accurate method that needs a germiculture period of 7 days followed by morphological, biochemical, and serologic confirmations (Bind et al., 1996). This method is labor intensive and time consuming, and has been replaced by molecular detection methods where rapid detection is required. Molecular detection methods developed for *L. monocytogenes* include PCR, quantitative PCR (qPCR), and multiplex qPCR (Long et al., 2008; Bickley et al., 2010; Chen et al., 2011; Witte et al., 2016). These methods shortened the detection time to several hours, but the dependence on laboratory equipment had limited their usage for on-site detections, especially in remote areas. Moreover, nowadays the supply chains in food industry are even longer and more complicated, which requires more affordable pathogen detection technologies to apply to the increasing food safety check points.

Advances of isothermal amplification techniques such as loop-mediated isothermal amplification of DNA (LAMP) and recombinase polymerase amplification (RPA) have provided molecular tools for pathogen detection that did not depend on laboratory equipment (Notomi et al., 2000; Piepenburg et al., 2006; Zhu et al., 2015). RPA is a promising method for its high sensitivity and good specificity. It needs a conveniently lower reaction temperature and fewer primer oligos compared to LAMP (Shi et al., 2015). The RPA reaction opens the two strands of the double-stranded DNA by the enzyme and amplifies the DNA target with the strand-displacing activity isothermally. DNA targets are exponentially amplified for detection within 20 min in a temperature range of 37–42°C (Liu et al., 2018). Using lateral flow strips (LFS) for end-point visual readout of the amplified DNA targets makes the method even less dependent on equipment (Cordray and Richardskortum, 2015; Yongkiettrakul et al., 2017). By using gold nanoparticles (AuNPs) specifically interacting with the labeled isothermal amplification products, colored signals are observed semi-quantitatively with the naked eye on LFS (Yang et al., 2013). Promising results from the RPA–LFS combined method have been reported for detection of *Staphylococcus aureus*, *Salmonella enteritidis*, and *L. monocytogenes* (Olsen et al., 1992; Lee et al., 2011; Gao et al., 2016; Wang et al., 2017; Wu et al., 2017; Du et al., 2018).

Nevertheless, false-positive signals from primer–dimers should be considered as an important intrinsic risk of the RPA–LFS combined method for pathogen detection. Primer–dimers are difficult to eliminate in DNA amplification reactions (Mendling et al., 2008). Unlike PCR and qPCR that have temperature cycles, primers mispairing to each other in the RPA reaction do not have the chance to dissociate and would certainly produce a fragment that can give a positive signal. Moreover, unlike electrophoresis or melting curve calculation that can indicate the fragment size, the LFS detection does not have the ability to distinguish the size of the molecule that gives a positive signal. Given the high sensitivity of the RPA–LFS combined method, interference of the positive signal by primer–dimers should be avoided.

In this study, a rapid and specific detection method for *L. monocytogenes* has been established. This RPA–LFS combined method eliminated false-positive signals from primer–dimers by careful design and rigorous screening of the optimal primer pairs on *L. monocytogenes* genome, and intelligent use of the probe to link the positive signal to the actual amplification product. This method finished the detection within 25 min and showed high specificity and sensitivity. It is a simple, rapid, and specific detection method for *L. monocytogenes* that has avoided the risk of false-positive results from primer–dimers.

## Materials and Methods

### Bacterial Strains and DNA Samples

*Listeria monocytogenes* (ATCC No. 19115), *Bacillus cereus* (ATCC No. 14579), *S. enteritidis* (ATCC No. 14028), *S. aureus* (ATCC No. 6538), *Escherichia coli* O157 (ATCC No. 43888), *Listeria grayi* (ATCC No. 25401), *Listeria innocua* (ATCC No. 33090), *Listeria seeligeri* (ATCC No. 35967), and *Listeria ivanovii* (ATCC No. 19119) at a concentration of 10^6^ colony-forming units (CFU)/ml in LB medium were kindly given from the Wuhan Institute for Food and Cosmetic Control (Wuhan, China). For PCR, qPCR, or RPA reactions, the cultures were heat treated at 100°C for 10 min before serving as the templates. If not specified, 1 μl of the heat-treated culture at 10^6^ CFU/ml was used as the template in these reactions. In experiments using the purified genomic DNA as the template, the genomic DNA was extracted and purified using a TIANamp Bacteria DNA Kit (Tiangen Biotech Co., Ltd., Beijing, China) from the bacteria culture as per the manufacturer’s instruction. The genomic DNA amounts were determined with a Qubit 4 fluorometer (Thermo Fisher Scientific) as per the manufacturer’s instruction.

### Primer BLAST

FASTA sequences of *L. monocytogenes* genome (GenBank No. CP025567.1; each input <50 kb) were entered into the NCBI Primer-BLAST software^1^. The product size was set as min at 300 and max at 500. The database was set as Refseq representative genomes. The organism was set as *L. monocytogenes* (taxid:1639). The primer size was set as min at 30 and max at 35. The primer GC content was set as min at 30 and max at 70. The max self-complementarity was set as any at 5′ and 3′ at 1. The max pair complementarity was set as any at 5′ and 3′ at 1. Other parameters were set as default.

### PCR Amplification and Electrophoresis

The PCR reaction contained 10 μl of Taq Mix (Monad Biotech Co., Ltd., Wuhan, China), 1 μl of each primer (10 μM; General Biosystems Co. Ltd., Anhui, China), 7 μl of distilled water, and 1 μl of the template. PCR cycles were set according to the manufacturer’s instruction of the PCR mix. Ten microliters of the PCR products were electrophoresed on a 2% agarose gel (Monad Biotech Co., Ltd., Wuhan, China).

### RPA Procedure

Recombinase polymerase amplification reactions were setup according to the manufacturer’s instructions of TwistAmp^®^ Liquid DNA Amplification Kit (TwistDx Inc., Maidenhead, United Kingdom). The reaction contained 25 μl of 2× reaction buffer, 5 μl of 10× Basic e-mix, 2.5 μl of 20× core mix, 2.1 μl of each primer (10 μM), 9.8 μl of distilled water, and 1 μl of the template. To initiate the reaction, 2.5 μl of magnesium acetate (280 mM) was added into the mixture. After a brief centrifugation, the reaction mixture was immediately incubated at 37°C for 30 min. The RPA amplification products were purified using the PCR Cleaning Kit (Monad Biotech Co., Ltd., Wuhan, China) and electrophoresed on a 2% agarose gel.

### Design of Probes

Probes were designed using the Primer Premier 3 software. The targeting sequences of the selected primer pairs were entered. The probe size was set as min at 46 and max at 53. The Tm was set as min at 57 and max at 70. The probe GC content was set as min at 30 and max at 80. The max hairpin score was set as 9. The max primer–dimer score was set as 9. The max poly-X was set as 5. Other parameters were set as default.

### RPA–LFS Procedure

The reverse primers and probes were modified with biotin and fluorescein isothiocyanate (FITC) at the 5′-ends, respectively (General Biosystems). The 3′-end of each probe was blocked with a C3-spacer, and a base “C” at the middle of the probe that was at least 30 bases away from the 5′-end and 15 bases away from the 3′-end was replaced with a [THF] group. RPA reactions were setup according to the manufacturer’s instructions of TwistAmp^®^ DNA Amplification nfo Kit (TwistDx). The reaction contained 29.5 μl of rehydration buffer, 2.1 μl of each primer (10 μM), 0.6 μl of probe (10 μM), 12.2 μl of distilled water, 1 μl of the template, and a dried enzyme pellet. To initiate the reaction, 2.5 μl of magnesium acetate (280 mM) was added into the mixture. After a brief centrifugation, the reaction mixture was immediately incubated at 30–45°C for 5–35 min. Five microliters of the amplification products was used for LFS (Ustar Biotechnologies Ltd., Hangzhou, China) detection. The amplification products were added to the sample pad of LFS, and the stick of LFS was inserted into 100 μl of the sample buffer (Ustar Biotechnologies Ltd., Hangzhou, China) for 2 min and then for visual reading.

### Preparation of *L. monocytogenes* Contaminated Milk

Raw milk purchased from the local market was aliquoted into 180-μl portions. Twenty microliters of the *L. monocytogenes* inactivated culture (10^6^ CFU/ml) was randomly mixed into some of the milk portions under room temperature. The milk portions served as samples for RPA–LFS and qPCR detection of *L. monocytogenes*.

### Quantitative PCR

A pair of specific primers (forward: 5′-TCCGCAAAAG ATGAAGTTC-3′; reverse: 5′-ACTCCTGGTGTTTCTCGATT-3′) targeting the hemolysin gene (*hlyA*) (GenBank No. M24199) of *L. monocytogenes* was used for qPCR (Jothikumar et al., 2003). The qPCR reaction mixture contained 10 μl of MonAmp^TM^ SYBR Green qPCR Mix (Monad Biotech Co., Ltd., Wuhan, China), 0.4 μM of each primer, 1 μl of the template, and 8.2 μl of distilled water. The cycling program was 95°C 30 s followed by 40 cycles of 95°C 10 s and 60°C 30 s on a Roche LightCycler 480 qPCR machine. The melting curve analysis was set as default.

### Preparation of Food Samples for RPA–LFS Evaluation

Ham, soft cheese, smoked fish, raw milk, pork, and beef samples were purchased from the local market and verified to be free of *L. monocytogenes* according to the National Standard of China (GB 4789.30-2016). One milliliter of 10 CFU/ml *L. monocytogenes* was added to the food samples (25 g or 25 ml, ground in liquid nitrogen if not liquid), and the contaminated food samples were mixed with 100 ml LB broth and incubated under 37°C with 250 rpm shaking for 3, 6, and 12 h. One microliter of the enrichment solution was collected, and DNA was extracted using the TIANamp Bacteria DNA Kit (Tiangen Biotech Co., Ltd., Beijing, China) for the RPA–LFS test.

## Results

### A Special Probe Could Reduce False-Positive Signals From Primer–Dimers

**FIGURE 1:**
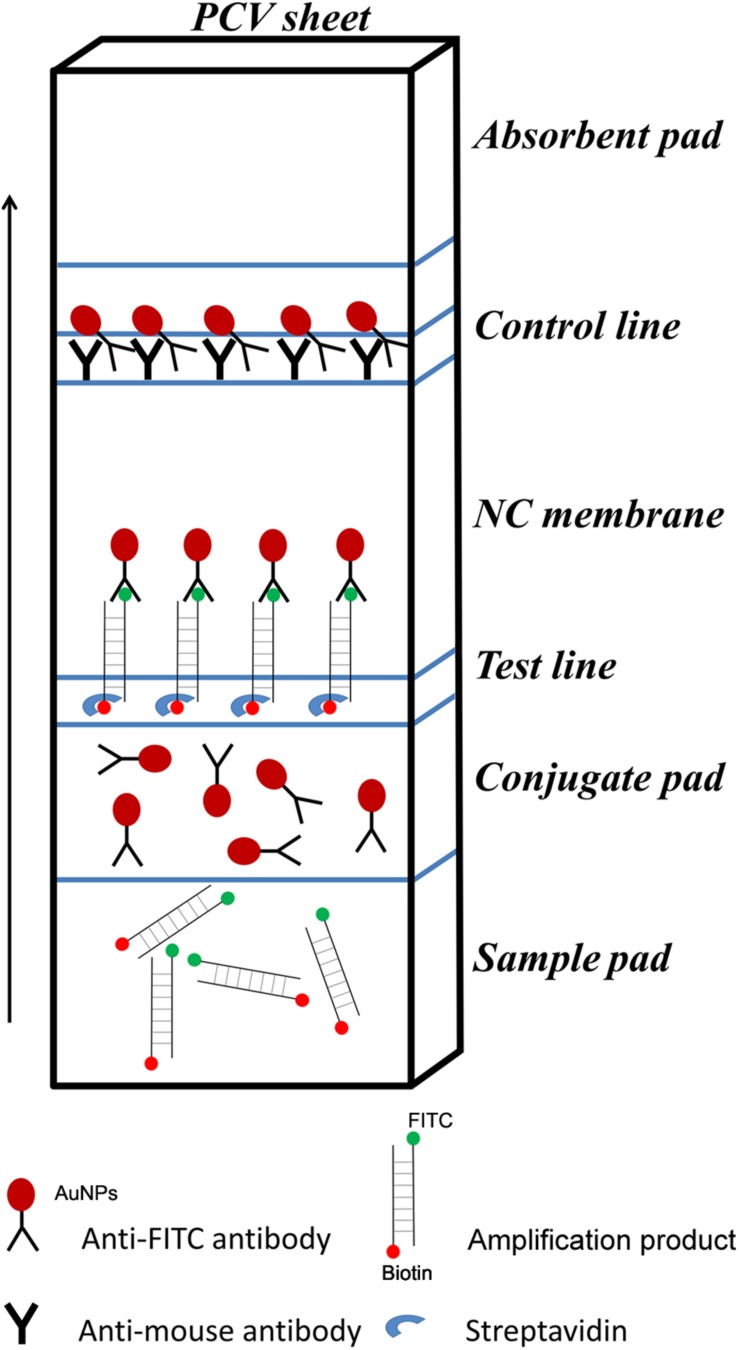
Schematic representation of the lateral flow strip (LFS) working principle. The borders of segments on the strip were indicated by blue lines. The names of the segments were indicated on the right of the strip drawing. The coating material on each segment of the strip was indicated by a shape. The liquid migration direction is indicated by an arrow. Molecules could be trapped by the materials on the Test line and the Control line, which were also indicated by different shapes. Shapes and their representing molecules were listed under the strip drawing.

For LFS detection, the control line is coated with anti-mouse antibody, and the test line on the strip is coated with streptavidin. The test line, which is closer to the sample pad, traps molecules labeled with biotin when the sample goes through. If the molecule labeled with biotin also labeled with FITC, it will bind to the AuNPs because the AuNPs are coated with anti-FITC antibody. This will make an aggregation of AuNPs at the test line to show a positive signal (red color). The control line, which is farther to the sample pad for validation of the LFS detection, only traps the AuNPs, because the anti-FITC antibody coated on the AuNPs is from mouse. Thus, the positive signal solely comes from molecules labeled with both biotin and FITC in the sample (Figure 1).

**FIGURE 2:**
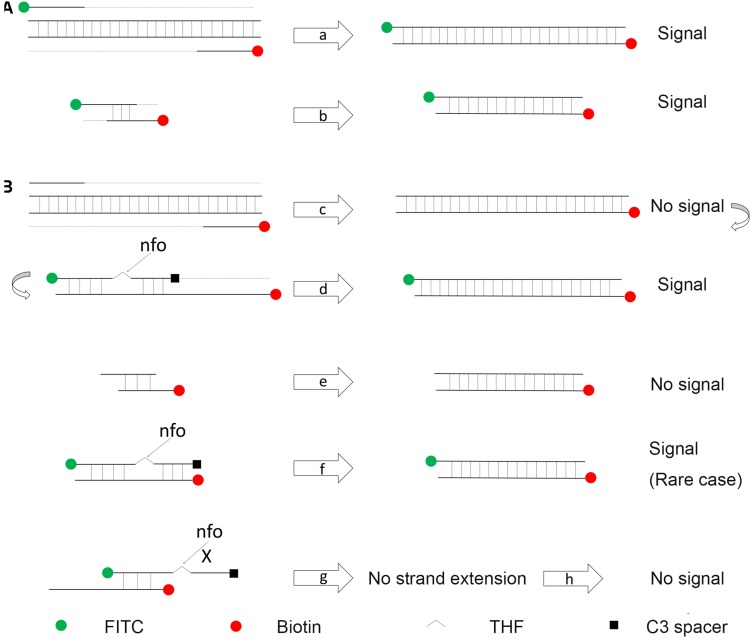
Schematic representation of a specially designed probe that reduces false-positive signals from primer–dimers. (A) In a typical RPA amplification, both the amplification products (a) and the primer–dimers (b) can give positive signals. (B) In the modified RPA reaction, amplification of the target DNA from the primer pair does not give the positive signal (c). This amplification product goes through another round of amplification guided by the probe to give a positive signal (d). Primer–dimers in the modified RPA reaction do not give the positive signal (e). Primer–probe complexes for most of the time do not give the positive signal (g). Only in a rare case that the primer and the probe have a good matching of the bases flanking the [THF] site, the complex can give the positive signal (f). DNA strands are presented as horizontal lines, and the base pairings are indicated as short vertical lines between the DNA strands. Anticipated amplification of the DNA strands is indicated as dotted lines. The nfo cleavage is indicated by arrows, and blocked cleavage is indicated by an “X.” Labels and modifications on DNA are indicated with different shapes and colors, with the legends given at the bottom of the figure.

In a typical RPA reaction, FITC and biotin are labeled at the 5′-end of each primer. The amplification product has both biotin and FITC labels, while the primer–dimers, if formed, also have the same labels that can give the positive signal (Figure 2A). In a modified RPA reaction, a specially designed probe will let only the amplification product to give positive signal (Figure 2B; Piepenburg et al., 2006). Instead of labeling FITC on the forward primer, FITC was labeled on the 5′-end of the probe. The 3′-end of the probe was labeled with a C3-spacer that could block the amplification. Also, a [THF] site was put in the middle of the probe for nfo cleavage. In this modified RPA reaction, amplification from the primer pair would only give the product with biotin labeled at one end. The probe completely matching one of the product strands would be cleaved by nfo at the [THF] site (nfo only works when the bases flanking the [THF] site are completely matching the other strand), freeing the 3′-end for elongation. This probe-guided amplification would give the positive signal product (Figure 2B, cases c,d). Primer–dimers would not give any signal (Figure 2B, case e). The partially paired probe–primer complex (case g) cannot be stabilized by strand extension from the 3′-end of the probe and would be only transient. Only in a rare case that the probe and the primer are matching multiple bases flanking the [THF] site (case f), there would be false-positive products. However, careful design and rigorous screening of probe and primers are still necessary to avoid this false-positive case; otherwise, just using a probe in RPA cannot guarantee the elimination of primer-dependent artifacts (Gao et al., 2016).

### Design and Screening of Primer–Probe Sets for the RPA–LFS System

**FIGURE 3:**
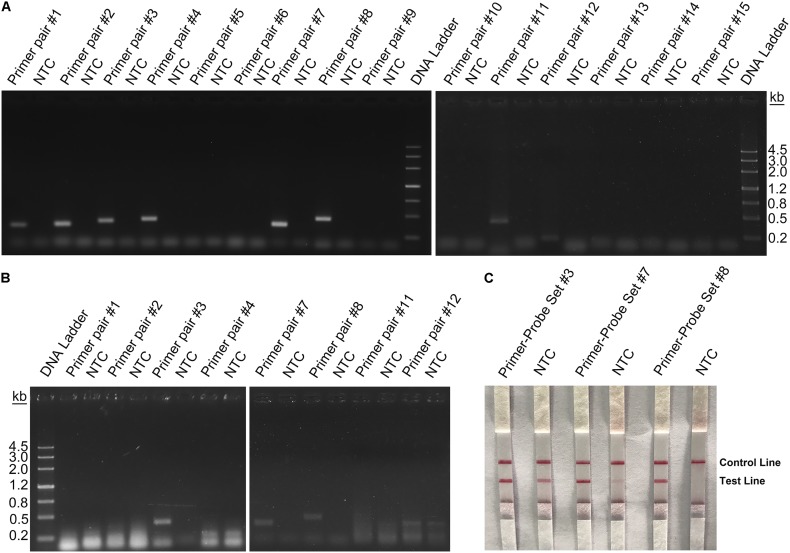
Screening of primers and probes. **(A)** Image of an agarose gel showing the PCR amplification of the primers using *L. monocytogenes* culture as the template. The primer pair name was indicated on top of each lane. The NTC lane immediately after is the no-template control of the respective primer pair. The band sizes of the DNA ladder are shown on the right. **(B)** Image of an agarose gel showing the RPA amplification of the primers using *L. monocytogenes* culture as the template. The primer pair name was indicated on top of each lane. The NTC lane immediately after is the no-template control of the respective primer pair. The band sizes of the DNA ladder are shown on the left. **(C)** The image shows that the LFS results of RPA amplifications with different primer–probe sets. The name of each primer–probe set is indicated on top of each strip. The NTC lanes are the no-template controls of the reactions. The positions of test and control lines are marked on the right of the strip image. The template was the *L. monocytogenes* culture. The reactions were performed at 40°C for 20 min.

Rational design of the primer–probe set for the RPA–LFS system started with design and selection of the primer pairs. Targeting the genome sequence of *L. monocytogenes* (GenBank No. CP025567.1), Primer BLAST was performed with the following criteria put into the parameter settings: (1) the primer pair should only target the species of interest (*L. monocytogenes*, taxid:1639) and (2) the primer pair should have less than five consecutive bases (and less than one if located at the 3′-end) pairing each other. Because one run of the Primer BLAST only covers ∼50 kb of the sequence, we started the Primer BLAST search from the 5′-end of the genome sequence for consecutive 50-kb areas. Some areas did not return any primer pair sequence. For areas that returned more than one primer pairs, we manually selected the primer pair that had the least number of bases pairing each other. We arbitrarily determined to collect 15 primer pairs from the Primer BLAST search to go on for the subsequent screening (Table 1). These 15 primer pairs targeting 15 different areas of the *L. monocytogenes* genome were first screened for their ability to amplify their targeting DNA fragments with normal PCR. Of these 15 primer pairs, 8 pairs gave positive results in the PCR amplification. These eight primer pairs were further tested for RPA amplification, and three of them gave positive results (Figures 3A,B).

**TABLE 1:** Design and screening of primer–probe sets for the RPA–LFS system.

Primer pair	Primer sequences	Targeting area (GenBank CP025567.1)	PCR	RPA	Probe sequence	RPA–LFS (normal/NTC)
1	TTTTGAGGAATACATATACTGGGATAAAAGAAG CAAGGAAACGTAGTAGGAAATCAAACAAAATAG	1473087 … 1473404	+	–	N/A	N/A
2	CGATTGTCGTAGTTATTGGTTCTTATTGGG CTTATTCCCTGACTATGTTTCAGGTCATTC	19875 … 20210	+	–	N/A	N/A
3	CTAGAAAAGTTCGTGTGGGTGATAAGATTG GTTTCGTTAATAAGTAAGCCGTTGGATAGG	834230 … 834609	+	+	FITC-GAACCAGAGGCAATTCATGTTATGCGTCTCG [THF]GGGAAACGGAGGAAG-/C3-spacer/	+/+
4	CTACCATGTTGCCAGTATTTGGATCTTTTC CAATGACAGTAGAAAAATGGAACGTAGACC	822187 … 822588	+	–	N/A	N/A
5	GATGTCAGTTTAGATGCAGATTTTACCGAG CATTGTCTCATTTTACTATTCTTTCCGCCC	845591 … 845946	–	N/A	N/A	N/A
6	GACCACTTCTTTCTGGGAAGTTAGATTTTG CTGGTTTTACGAAGACAGGATATGTAAGGA	891248 … 891661	–	N/A	N/A	N/A
7	ATAGAATAGGAACCCTGCTACTAAAATCGG CAAGTAACATAGAAACACCTCTCCTTCAAC	943573 … 943893	+	+	FITC-GAAATCGTGAAAACTTCAAATTCTCCCCTTC [THF]TTTCTAAAACCTTAT-/C3-spacer/	+/+
8	CTCGCTCTAGGTATCTTTGGGATTTACTAC GTCCGAATATCATTTACCTCATCAAAAGGG	985348 … 985769	+	+	FITC-GTTTCATTCCTGCGTTACTATTCATTGTTG [THF]TGCTATACTTTGTTT-/C3-spacer/	+/−
9	GATTTGAACATCCAGTAATTGAAGAGCGAG GGTTATCATCTCACCTAAATAAACCCACGA	969562 … 969941	–	N/A	N/A	N/A
10	CCAAGTAATGACAGTAAAAGAAATCAAAGATAC TAATGCAGGTAAATCATCTAAAGAGACAACAAC	1046654 … 1046954	–	N/A	N/A	N/A
11	GTAAAAGATAAATAAATTGCTAATAGGTGGGGA GTTATTACTTCTTCTTCAAGCAACCTCAAA	248100 … 248536	+	–	N/A	N/A
12	GTAAGTGGGAAATCTGTCTCAGGTGATGTAG ACTCCTGGTGTTTCTCGATTAAAAGTAGCA	213843 … 214023	+	–	N/A	N/A
13	GGAAAAGCAAGAAATATCAAGTCGAAGTAG CATTATACATAGTCAAAAACATACCGTCAATC	262260 … 261901	–	–	N/A	N/A
14	CGATTTATCTTATGTTACTGGTGTGGATTT GTAATTGTTTAGCTGATTTATAGGCTTTTGG	272084 … 271624	–	–	N/A	N/A
15	GCTAGTATAAAGAGATGGATTAGTTTTCTGG AAATTGTTGTAAATCTTCTAGTTCGTCTGGTG	267536 … 267139	–	–	N/A	N/A

*“+” means positive amplification or signal, “−” means no amplification or negative signal, N/A, not applicable, “Normal” means the *Listeria monocytogenes* culture template and all other reaction components were given normally for reaction, “NTC” means the no-template control. RPA, recombinase polymerase amplification; LFS, lateral flow strip.*

**FIGURE 4:**
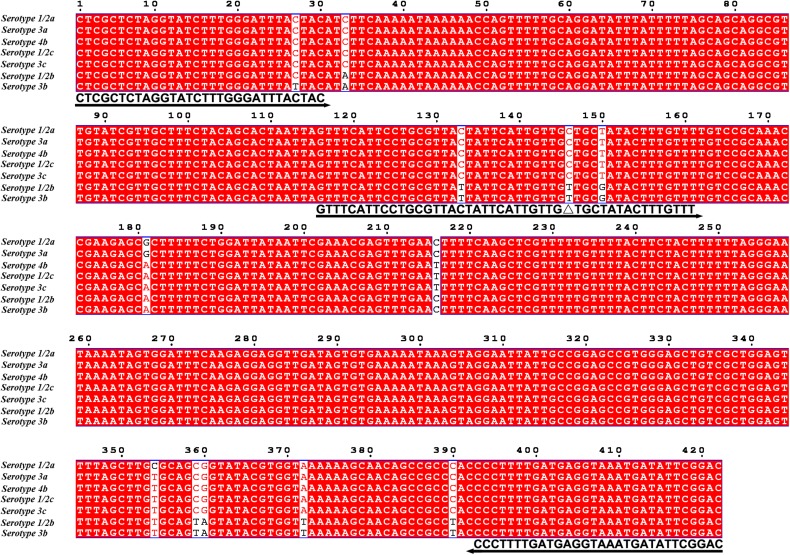
Targeting fragment of the primer–probe set #8. The alignment of the targeted DNA fragments from seven different serotypes of *L. monocytogenes* was performed by NCBI BLAST. The serotypes are indicated at the beginning of each sequence. The sequences corresponding to the primers and the probe are written under their positions in the alignment. The arrow lines indicate the direction of extension of the primers and probe. The [THF] site is represented by a “Δ.” Serotype 4b is corresponding to genome sequence of GenBank No. CP025567.1.

Because primer–dimer bands were observed on the gels for both PCR and RPA amplifications, a probe within the respective targeting fragment was designed for each of the three primer pairs that had specific RPA amplifications (Table 1). Parameters were set so that there were less than five consecutive bases in the probe that could pair to the respective primer pair. The primer-probe sets were screened in an RPA–LFS process, and only one set (#8) gave the correct positive signal (two visible pink bands at both the Test and the Control Lines); meanwhile, the false-positive signal was avoided (only one visible pink band at the Control Line for the no-template control) (Figure 3C). The DNA fragment targeted by primer-probe set #8 was 422 bp in length, and this fragment is conserved within the major pathogenic serotypes of *L. monocytogenes*, including serotypes 4b, 1/2a, 1/2b, 1/2c, 3a, 3b, and 3c (Figure 4). The primer pair in set #8 was checked by NCBI Nucleotide BLAST, and the 100 returned hits were all *L. monocytogenes* sequences. No sequences of other species were returned (Supplementary File S1). This means the primer-probe set #8 would be able to detect all the major pathogenic serotypes and should be highly specific. Since ∼90% of the global cases of illness related to *L. monocytogenes* were from serotypes 4b, 1/2a, 1/2b, and 1/2c (Braga et al., 2017; Datta and Burall, 2018), the primer-probe set #8 would be able to cover >90% of the detection needs. The primer-probe set #8 was used for all the following RPA–LFS reactions in this study.

### Optimization of the RPA–LFS Conditions

**FIGURE 5:**
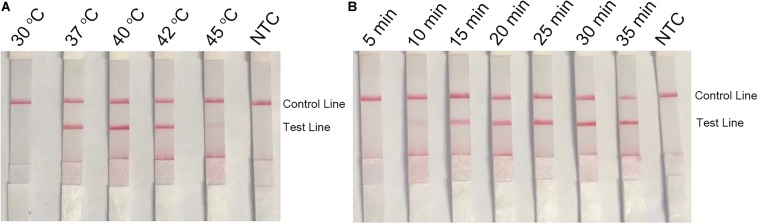
Optimal reaction temperature and time of the RPA–LFS system. **(A)** The image shows the LFS results of RPA amplifications under different temperatures. The temperatures under which the RPA reactions were performed are indicated on top of each strip. The amplification template was *L. monocytogenes* culture. The NTC strip is the no-template control that performed at 40°C. **(B)** The image shows the LFS results of RPA amplifications with different time lengths. The time lengths for which the RPA reactions were performed are indicated on top of each strip. The amplification template was *L. monocytogenes* culture. The NTC strip is the no-template control that performed for 25 min. The positions of the Control and Test lines are indicated on the right of the images.

To optimize the reaction temperature of the RPA–LFS system, the RPA assay was performed at temperatures ranging from 30 to 45°C. The reaction time was set at 30 min, and the amplification results were analyzed by LFS. The pink band at the Test Line was visible at temperatures 37, 40, and 42°C, and was most visible at 40°C (Figure 5A). Furthermore, the RPA reaction time was screened from 5 to 35 min. The pink band at the Test Line appeared at 10 min and became darker from 15 min. After 20 min, the darkness of the band did not change significantly (Figure 5B). Thus, 40°C and 20 min were selected as the optimal reaction temperature and time for the RPA procedure.

### Detection Specificity of the RPA–LFS System

**FIGURE 6:**
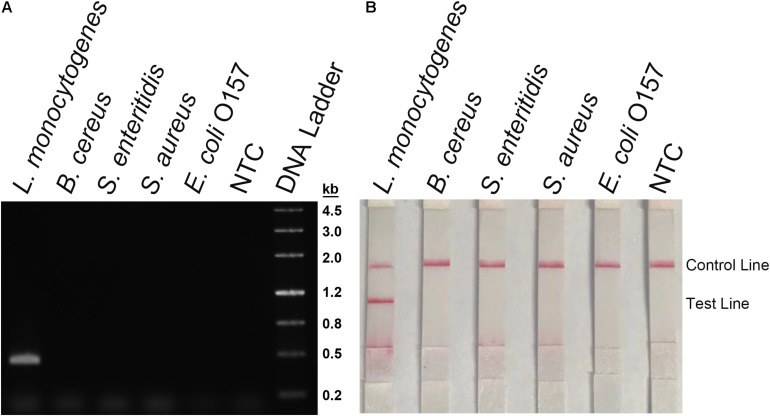
Detection specificity among commonly seen foodborne pathogens. **(A)** An agarose gel image showing the RPA amplification results from different bacterial culture templates. The species of the bacteria are indicated on top of each lane. The NTC lane is the no-template control. The size of each band of the DNA ladder is indicated on the right of the gel image. **(B)** Image of the LFS results of RPA amplification of different bacterial culture templates. The species of the bacteria are indicated on top of each strip. The NTC strip is the no-template control. The positions of the Control and Test lines are indicated on the right of the image. The reactions were performed at 40°C for 20 min.

During the primer design process, parameters had been set to target only *L. monocytogenes* but not any species else. To confirm the specificity of the primer-probe set, the primer pair was tested for RPA amplification of four other pathogenic bacterial species that were commonly tested for food contaminations, namely, *B. cereus*, *S. enteritidis*, *S. aureus*, and *E. coli* O157. Using culture solutions of these bacteria as the template, RPA with the primer pair showed no amplification on the agarose gel, while using the *L. monocytogenes* culture as the template showed a clear and specific amplification band (Figure 6A). These bacterial cultures were also tested in the RPA–LFS system as the templates. Only the *L. monocytogenes* culture showed a positive result, and the other bacterial cultures were negative (Figure 6B). This indicated that the primer-probe set had a good specificity toward *L. monocytogenes* and would not cross-react with the four other pathogenic bacteria.

**FIGURE 7:**
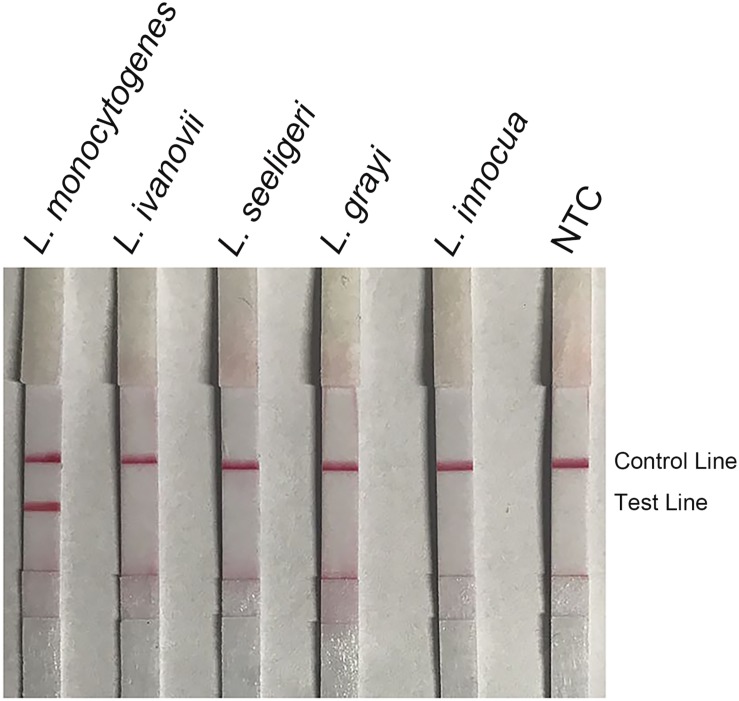
Detection specificity among non-pathogenic *Listeria* species. Image of the LFS results of RPA amplification of different bacterial culture templates. The species of the bacteria are indicated on top of each strip. The NTC strip is the no-template control. The positions of the Control and Test lines are indicated on the right of the image. The reactions were performed at 40°C for 20 min.

The non-pathogenic *Listeria* species including *L. grayi*, *L. innocua*, *L. seeligeri*, and *L. ivanovii* were used as templates in the RPA–LFS system (Figure 7). All the non-pathogenic *Listeria* species were negative in the test, suggesting that the system would detect only *L. monocytogenes* but not the non-pathogenic strains.

### Detection Limit of the RPA–LFS System for *L. monocytogenes*

**FIGURE 8:**
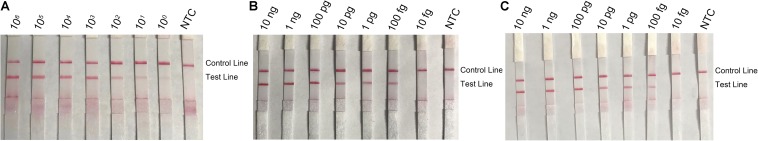
Detection limit of the RPA–LFS system. **(A)** The image shows the LFS results of RPA amplifications with different amounts of *L. monocytogenes* culture. The amounts (in CFU) added to the RPA reactions are indicated on top of each strip. **(B,C)** Images of the LFS results of RPA amplifications with different amounts of *L. monocytogenes* genomic DNA. The amounts added to the RPA reactions are indicated on top of each strip. In panel **C**, 10 ng of the genomic DNA of *B. cereus* was added to the reactions in addition to the *L. monocytogenes* genomic DNA. The NTC strips are the no-template controls. The reactions were performed at 40°C for 20 min. The positions of the Control and Test lines are indicated on the right of the images.

To determine the detection limit of the RPA–LFS system for *L. monocytogenes*, a 10-fold series dilution of inactivated *L. monocytogenes* culture ranging from 10^0^ to 10^6^ CFU μl^–1^ were tested (reaction volume: 50 μl; 1 μl of the diluted culture was added into each reaction). The results showed that, although weak, a pink band still appeared at the Test Line with 10^0^ CFU/μl. Also, the pink band darkened with the increasing concentrations of *L. monocytogenes* (Figure 8A). In a similar setting, 10-fold series dilutions of purified *L. monocytogenes* genomic DNA were tested. As low as 100 fg of the *L. monocytogenes* genomic DNA could be detected (Figure 8B). To test if the system could resist the interference of other bacterial DNA, 10 ng of the genomic DNA of another commonly seen foodborne pathogen, *B. cereus*, was added into the RPA reactions along with the dilutions of *L. monocytogenes* genomic DNA. The *B. cereus* genomic DNA did not interfere with the detection of *L. monocytogenes* (Figure 8C). We concluded that the detection limit of the RPA–LFS system was 1 CFU per reaction without DNA purification, or 100 fg of genomic DNA/50 μl. The detection sensitivity was not affected by other bacterial DNA.

### Application of the RPA–LFS System for *L. monocytogenes* Detection in Food Samples

The RPA–LFS system was applied to the detection of *L. monocytogenes* in contaminated raw milk samples, and the performance was compared with the traditional quantitative PCR method. Forty-eight milk samples were prepared, with four of them contaminated with *L. monocytogenes*. The 48 samples were randomly numbered and were subject for detection of *L. monocytogenes* with both RPA–LFS and qPCR. For judging the quantitative PCR results, we considered a Ct value of <32 as the positive contamination. All the four contaminated samples were successfully detected, and the results of the RPA–LFS system were consistent with those from quantitative PCR (Table 2).

**TABLE 2:** Detection performance of the RPA–LFS system and quantitative PCR.

No.	RPA–LFS	Quantitative PCR		No.	RPA–LFS	Quantitative PCR		No.	RPA–LFS	Quantitative PCR	
		Result	Ct value (*n* = 3)			Result	Ct value (*n* = 3)			Result	Ct value (*n* = 3)
1	−	−	36.71 ± 0.15	17	−	−	37.13 ± 0.34	33	−	−	36.13 ± 0.25
2	−	−	36.70 ± 0.25	18	−	−	37.69 ± 0.36	34	−	−	37.54 ± 0.44
3	−	−	37.18 ± 0.05	19	−	−	36.90 ± 0.27	35	−	−	33.14 ± 0.23
4	−	−	38.70 ± 0.18	20	−	−	35.96 ± 0.32	36	−	−	37.19 ± 0.30
5	−	−	37.20 ± 0.34	21	+	+	23.45 ± 0.21	37	−	−	36.79 ± 0.45
6	−	−	35.92 ± 0.26	22	+	+	23.29 ± 0.45	38	−	−	37.30 ± 0.15
7	−	−	37.08 ± 0.06	23	−	−	36.45 ± 0.35	39	−	−	36.75 ± 0.35
8	−	−	36.13 ± 0.19	24	−	−	37.05 ± 0.15	40	−	−	36.93 ± 0.24
9	−	−	37.05 ± 0.17	25	−	−	37.54 ± 0.11	41	−	−	32.9 ± 0.18
10	−	−	33.14 ± 0.12	26	−	−	36.71 ± 0.12	42	−	−	37.13 ± 0.15
11	−	−	37.19 ± 0.45	27	−	−	36.70 ± 0.19	43	−	−	36.94 ± 0.32
12	−	−	36.79 ± 0.46	28	−	−	37.18 ± 0.16	44	−	−	36.9 ± 0.22
13	−	−	38.20 ± 0.25	29	−	−	37.39 ± 0.15	45	−	−	35.96 ± 0.45
14	−	−	38.38 ± 0.13	30	−	−	37.20 ± 0.33	46	+	+	23.45 ± 0.15
15	−	−	38.47 ± 0.14	31	−	−	35.92 ± 0.15	47	+	+	23.29 ± 0.12
16	−	−	32.90 ± 0.19	32	−	−	37.08 ± 0.35	48	−	−	36.45 ± 0.17

*“+” means positive, “−” means negative.*

**FIGURE 9:**
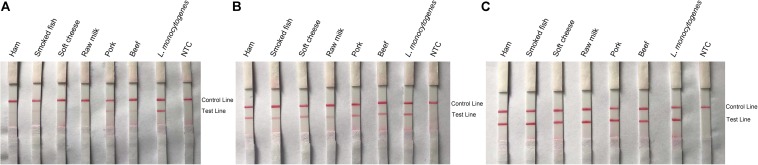
Detection of *L. monocytogenes* in spiked food samples. Images of the LFS results of RPA amplification of various food samples spiked with *L. monocytogenes* and enriched for 3 **(A)**, 6 **(B)**, and 12 h **(C)**. The food samples are indicated on top of each strip. The *L. monocytogenes* culture was used as the positive controls. The NTC strips are the no-template controls. The positions of the Control and Test lines are indicated on the right of the images. The reactions were performed at 40°C for 20 min.

Other food samples including ham, soft cheese, smoked fish, raw milk, pork, and beef (25 g or 25 ml) were spiked with 10 CFU of *L. monocytogenes* and enriched for 3, 6, and 12 h. DNA was extracted and purified from the enriched food samples and tested with the RPA–LFS system (Figure 9). After 6 h of enrichment, *L. monocytogenes* could be detected with distinct signal bands, and the signals became stronger after 12 h of enrichment.

## Discussion

Being an important foodborne pathogen and an explicit public health hazard, control of the spread of *L. monocytogenes* is an absolute requirement for food safety (Liu, 2006). Conventional biochemical identification and more recent PCR-based molecular detection methods are dependent on laboratory equipment and trained personnel, and cannot fulfill the nowadays requirement for affordable, specific, and sensitive detection methods to apply to the many and increasing food safety check points (Khueankhancharoen et al., 2016). The isothermal amplification method, RPA, is promising because of its fast amplification of the target DNA, the low isothermal conditions, and the ability to tolerate unpurified samples (Kersting et al., 2014). These features endow this method with speed, sensitivity, and simplicity. The chemical labeling of the RPA reaction enables the usage of AuNP-based LFS for the result readout, making the dependency on equipment, and trained personnel even lower. However, all these simplifications make the intrinsic risk of false-positive signals from primer–dimers non-negligible. Primer–dimers are very difficult to eliminate in DNA amplification reactions, and the hot-start strategies to reduce primer–dimer formation in PCR reactions are not applicable to the RPA system (Zachgo et al., 1995; Yuan et al., 2000). In the RPA reaction, mispaired primers cannot dissociate and are fated to produce signal-giving products. The LFS does not distinguish the size of signal-giving molecules and takes every such molecule as a positive signal. Since the method is very sensitive to detect as low as 1 CFU of *L. monocytogenes* per reaction, a very low amount of primer–dimers is a significant risk of false-positive results.

It has been reported that introducing a probe into the RPA reaction could reduce the primer-dependent artifacts (Piepenburg et al., 2006), but elimination of the false-positive signal was not guaranteed by just a probe (Figure 2B). We tried the previously reported primer pair and primer-probe set for RPA detection of *L. monocytogenes* (Gao et al., 2016; Du et al., 2018), and the results suggested that none of them completely eliminated the primer-dependent artifacts (Supplementary Table S1 and Supplementary Figure S1). Primer–dimer (or in this RPA case, primer–probe complex) formation is affected by many factors such as buffer contents, environment temperature, and mixture impurities (Atkin et al., 2010). Considering that the detection method is to be applied for food safety tests in variant conditions with unpurified samples and often operated by people with limited training, it has to be developed for a wide adaptability.

In this study, the primer–probe set was finalized with a rigorous selection and screening procedure. We did an extensive search for primer sequences on *L. monocytogenes* genome and limited the theoretical primer pairing chance to the lowest by setting strict parameters. The primer candidates were screened for specific amplification in PCR and RPA reactions. The probes were also designed with strict parameters, and finally, the primer–probe set #8 was obtained that avoided the false-positive signal (Table 1). Cross-dimer analysis of the primer–probe set RPA-1-RP (Gao et al., 2016) suggested 15 possible cross-dimers between the probe and the reverse primer, in which two cross-dimers fell in the rare case of false-positive signal (Figure 2B, case f). In contrast, the primer–probe set #8 from this study had eight possible cross-dimers between the probe and the reverse primer, but none was in the rare case of false-positive signal (Supplementary Figure S2). This could explain the unstable performance of the primer–probe set RPA-1-RP on false-positive signals, and also suggested that the primer–probe set #8 from this study should have completely eliminated the primer-dependent artifacts. Indeed, using the primer–probe set #8 in RPA–LFS did not give any false-positive signal in all of our tests.

Targeting the virulence genes is a common practice in molecular detection of pathogenic bacteria (Han and Wei, 2018). In the previously reported detection of *L. monocytogenes* with RPA, the *hlyA* gene was the target (Gao et al., 2016; Du et al., 2018). This ensured the detection specificity to the pathogenic strains but, on the other hand, limited the pool size of primer candidates. In this study, we selected primer candidates from the entire genome but not any particular gene. With strict parameter settings and careful design, our detection method showed superb specificity to all the major pathogenic serotypes of *L. monocytogenes* and avoided cross-reaction with other commonly seen foodborne pathogens or non-pathogenic *Listeria* species. Importantly, this strategy gave us a primer candidate pool that was large enough to screen for a primer–probe set that eliminated the primer-dependent artifacts.

Our RPA–LFS system for *L. monocytogenes* detection retained the good properties of RPA and LFS technologies. It was highly specific to *L. monocytogenes* and was able to detect as low as 1 CFU of the bacterium per reaction (50 μl) without DNA purification, or 100 fg of the genomic DNA/50 μl. This sensitivity was higher than the LAMP testing limit of 10^3^ CFU/ml and was comparable to the detection limit of PCR- or qPCR-based methods, which was between 10^2^ and 10^3^ CFU/ml (Ye et al., 2015; Jayanth and Varadaraj, 2017; Garrido-Maestu et al., 2018). The amplification could be conducted under the temperature between 37 and 42°C, and the whole detection finished within 25 min. In an application simulation, we tried the system with artificially contaminated milk samples. Direct testing without purification of the samples gave 100% accuracy of detection, which was consistent with the traditional qPCR method. Various food samples spiked with 10 CFU of *L. monocytogenes* per 25 g or 25 ml were successfully detected after an enrichment time period of 6 h. Our RPA–LFS system provided an improved *L. monocytogenes* detection method with high specificity, good sensitivity, and little dependency on equipment.

## Conclusion

In this study, we developed an improved RPA–LFS system for detection of *L. monocytogenes*. By careful design and rigorous screening of the optimal primer pairs on *L. monocytogenes* genome and intelligent use of the probe to link the positive signal to the actual amplification product, the system provided a rapid, simple, and specific detection method for *L. monocytogenes* that eliminated false-positive results from primer–dimers. Reducing equipment dependency of pathogen detection technologies is a challenging task of this era, and application of RPA and LFS is a promising direction. This study has set a good example of reducing an intrinsic and non-negligible risk of RPA and LFS, which is valuable for the development of detection technologies of other public health threatening species.

## Data Availability Statement

All datasets generated for this study are included in the article/Supplementary Material.

## Author Contributions

KZ, SG, and JD designed the research. LW, PZ, XS, JL, and XD conducted the research. PZ, SG, and JD wrote the manuscript. JD directed the project.

## Conflict of Interest

The authors declare that the research was conducted in the absence of any commercial or financial relationships that could be construed as a potential conflict of interest.

## Footnotes

1 https://www.ncbi.nlm.nih.gov/tools/primer-blast
