# Supplementary material for: Rapid and Specific Detection of Listeria monocytogenes With an Isothermal Amplification and Lateral Flow Strip Combined Method That Eliminates False-Positive Signals From Primer–Dimers
Source: Front Microbiol. 2020 Feb 6;10:2959. doi: 10.3389/fmicb.2019.02959 (PMC7025549; doi:10.3389/fmicb.2019.02959)
Supplement: Supplementary file 1 [file Data_Sheet_1.PDF]

Supplementary Tables and Figures

Table S1 Test of previously reported primer pair and primer-probe set for false positive signals

Primer Pair/ Primer-Probe Set	RPA-1-RP (Gao et al.,2016)		The primer pair for lateral flow RPA reaction (Du et al.,2018)		Primer-Probe Set #8 (from this study)	
	Normal	NTC	Normal	NTC	Normal	NTC
Trial #1	+	+	+	+	+	-
Trial #2	+	-	+	+	+	-
Trial #3	+	-	+	+	+	-
Trial #4	+	+	+	-	+	-
Trial #5	+	-	+	+	+	-

(Results of five independent test trials of the primer pairs/primer-probe sets in the RPA-LFS system are shown. "Normal" means the *Listeria monocytogenes* culture template and all other reaction components were given normally for reaction. "NTC" means the no-template control. "+" means the positive signal. "-" means the negative signal.)

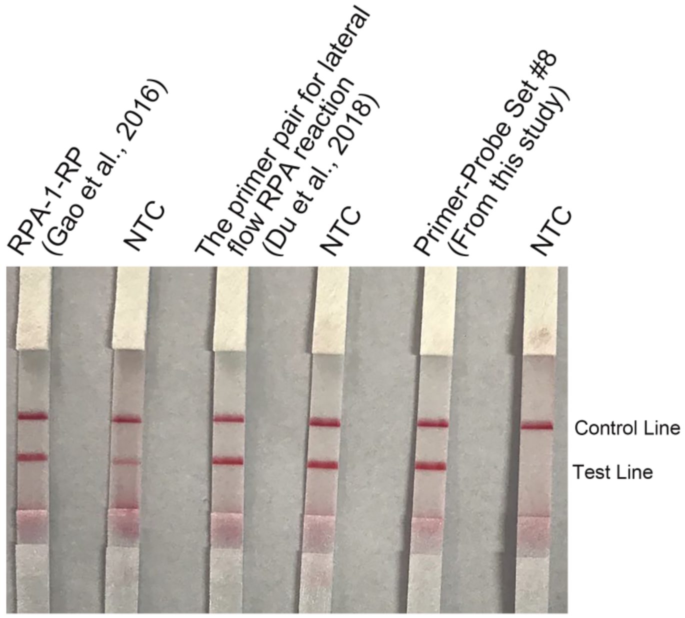

Figure S1 Test of previously reported primer pair and primer-probe set.

The image shows the LFS results of RPA amplifications with different primer pair or primer-probe sets from Trial #1 (**Table S1**). The name of each primer pair or primer-probe set is indicated on top of each strip. The NTC lanes are the no-template controls of the reactions. The positions of test and control lines are marked on the right of the strip image. The template was the *L. monocytogenes* culture. The reactions were performed at 40°C for 20 min.

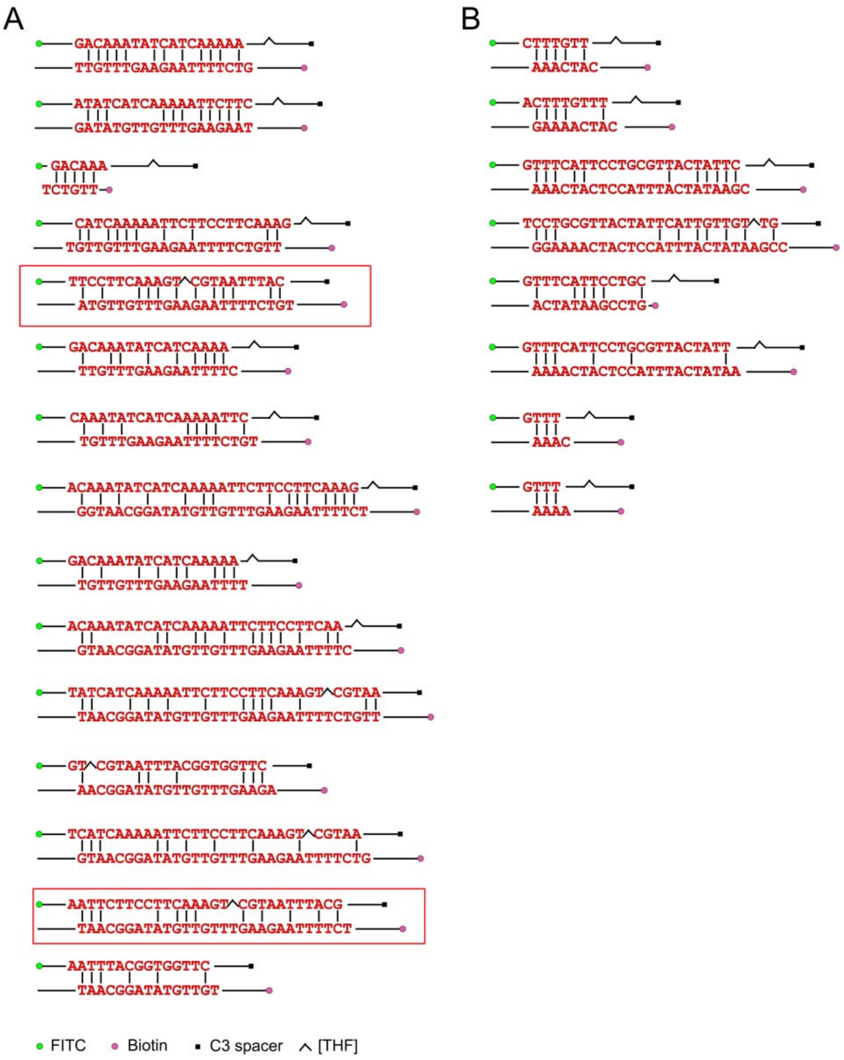

Figure S2 Cross-dimer analyses.

(A) Cross-dimer analysis of the primer-probe set RPA-1-RP (Gao et al.,2016) suggested 15 possible cross-dimers between the probe and the reverse primer. (B) Cross-dimer analysis of the primer-probe set #8 from this study suggested 8 possible cross-dimers between the probe and the reverse primer. Cross-dimers that could lead to false positive signals are indicated with red boxes. Base pairings are indicated as short vertical lines between the DNA strands. Labels and modifications on DNA are indicated with different shapes and colors, with the legends given at the bottom of the figure. The analyses were done with the Primer Premier 5 software.

Supplementary References

Gao, W., H. Huang, Y. Zhang, P. Zhu, X. Yan, J. Fan and X. Chen (2016). Recombinase Polymerase Amplification-Based Assay for Rapid Detection of Listeria monocytogenes in Food Samples. Food Analytical Methods 10(6): 1972-1981. doi: 10.1007/s12161-016-0775-0

Du, X. J., Y. X. Zang, H. B. Liu, P. Li and S. Wang (2018). Recombinase Polymerase Amplification Combined with Lateral Flow Strip for Listeria monocytogenes Detection in Food. J Food Sci 83(4): 1041-1047. doi: 10.1111/1750-3841.14078
